# Predicting Phrenic Nerve Palsy in Patients Undergoing Atrial Fibrillation Ablation With the Cryoballoon—Does Sex Matter?

**DOI:** 10.3389/fcvm.2021.746820

**Published:** 2021-12-14

**Authors:** Alexander Pott, Hagen Wirth, Yannick Teumer, Karolina Weinmann, Michael Baumhardt, Christiane Schweizer, Sinisa Markovic, Dominik Buckert, Carlo Bothner, Wolfgang Rottbauer, Tillman Dahme

**Affiliations:** Department of Medicine II, Ulm University Medical Center, Ulm, Germany

**Keywords:** cryoballoon, phrenic nerve palsy, ablation, atrial fibrillation, female gender

## Abstract

**Background:** Phrenicus nerve palsy (PNP) is a typical complication during pulmonary vein isolation (PVI) using the cryoballoon with the ominous potential to counteract the clinical benefit of restored sinus rhythm. According to current evidence incidence of PNP is about 5–10% of patients undergoing Cryo-PVI and is more frequent during ablation of the RSPV compared to the RIPV. However, information on patient specific characteristics predicting PNP and long-term outcome of patients suffering from this adverse event is sparse.

**Aim of the Study:** To evaluate procedural and clinical characteristics of AF patients with PNP during cryoballoon PVI compared to patients without PNP.

**Methods and Results:** Between 2013 and 2019 we included 632 consecutive AF patients undergoing PVI with the cryoballoon in our study. 84/632 (13.3%) patients experienced a total number of 89 PNP during the ablation procedure. 75/89 (84%) cryothermal induced PNP recovered until the end of the procedure (transient PNP, tPNP), whereas 14/89 (16%) PNP hold beyond the end of the procedure (non-transient PNP, ntPNP). Using multivariate logistic regression, we found that sex and BMI are strong and independent predictors of cryothermal induced non-transient PNP during cryoballoon PVI with an odds ratio of 3.9 (CI: 95%, 1.1–14.8, *p* = 0.04) for female gender. Interestingly, all patients (14/14, 100%) with a non-transient PNP experienced complete PNP resolution after a mean recovery time of 68 ± 79 days.

**Conclusion:** Our data indicate for the first time, that female sex and lower BMI are independent predictors for non-transient PNP caused by cryoballoon PVI. Fortunately, during follow up all PNP patients resolved completely with a median recovery time of 35 days.

## Introduction

Based on its clinical efficiency and procedural safety cryoballoon pulmonary vein isolation (CB-PVI) has been established as an important therapeutic option for the treatment of patients suffering from atrial fibrillation (AF) (1–4). Severe procedural complications are sparse in patients undergoing CB-PVI with phrenic nerve palsy (PNP) during cryoenergy application of the right-sided pulmonary veins (PV) as the most frequent adverse event (5–7).

The right phrenic nerve (rPN) arises from the right cervical plexus and is located between the right-sided pericardium and the mediastinal pleura at the anterolateral wall of the superior vena cava (SVC) (8). Due to the anatomical proximity between SVC and right sided pulmonary veins rPN is frequently exposed to ablation energy during pulmonary vein isolation (PVI), especially when cryoenergy is applied. In case of phrenic nerve injury diaphragm movement can be limited or even abolished leading to severe dyspnoea counteracting the clinical benefit of restored sinus rhythm (9).

Various data have been published regarding incidence, outcome and clinical impact of PNP depending on ablation technique, balloon size, monitoring of PN activity and PNP definition (10–13). However, patient specific parameters predicting PNP occurrence during cryoballoon-PVI is rather sparse limiting pre-procedural risk evaluation and individualized informed consent of (AF) patients undergoing ablation (14, 15). Furthermore, data on long-term clinical outcome of cryoenergy-induced PNP and its recovery rate is only little studied so far (13).

Hence, the aim of our study was to compare clinical characteristics of AF patients with PNP during cryoballoon PVI compared to patients without PNP and to identify patient specific parameters predicting PNP occurrence. Furthermore, we assessed long-term clinical course of patients suffering from PNP which holds beyond the end of the ablation procedure.

## Methods

### Study Population

Between 2013 and 2019 we included 632 consecutive patients undergoing PVI with the 2nd or 3rd generation cryoballoon in this observational study. From 2016 patients were routinely included in our prospective arrhythmia registry ATRIUM (ATrial Fibrillation Registry and BIobank UlM, DRKS-ID: DRKS00013013). Exclusion criteria of ATRIUM are the following:

- Age < 18 years
- permanent atrial fibrillation
- LA > 60 mm
- unable to consent

Beyond these exclusion criteria patients with pre-existing right-sided PNP were retrospectively excluded from our study. Patients with phrenic nerve palsy that occurred during cryoballoon PVI were assigned to the PNP group. PNP that restored within in the ablation procedure was classified as transient PNP (tPNP), whereas PNP that remained beyond the end of the procedure was classified as non-transient PNP (ntPNP). The study complies with the Declaration of Helsinki and was approved by our institutional review committee (342/16 and 05/2017). All patients gave written informed consent to the procedure.

### Aim of the Study

To evaluate procedural and clinical characteristics of AF patients experiencing either transient or non-transient PNP during cryoballoon PVI compared to patients without PNP and to assess rate of PNP reconstitution during long-term follow-up.

### Periprocedural Management

Intracardiac thrombi were ruled out in every patient by transesophageal echocardiography prior to PVI. No additional pre-procedural imaging was applied. All patients underwent transthoracic echocardiography to rule out pericardial effusion immediately after the procedure and prior to hospital discharge. Periprocedural anticoagulation was conducted as described before (16).

### Cryoballoon Ablation Procedure

Cryoballoon ablation procedure was performed either 2nd or 4th generation cryoballoon and has been described in detail before (17). Briefly, a steerable sheeth (Medtronic, Minneapolis, MN, Flexcath Advance) was used to introduce the cryoballoon in the left atrium after single transseptal puncture. After balloon inflation at the PV ostia a 20-mm spiral mapping catheter (Achieve, Medtronic, Minneapolis, MN) was positioned in the PV at the closest achievable proximity to enable real-time observation of PV potentials during PV isolation. PV occlusion was documented by injection of contrast medium.

Phrenic nerve integrity was monitored by stimulation *via* the diagnostic catheter placed in the superior vena cava and palpation of the right-sided diaphragm and with additional recording of compound motor action potentials (CMAP) of the right sided diaphragm (18, 19). Cryoenergy application was aborted in case of reduced palpational feedback and/or reduced CMAP. In general, cryoenergy application was stopped by a single-stop-technique.

### Post-procedural Management and Clinical Follow-Up

In patients with ntPNP ultrasound examination and/or x-ray of the chest was performed to evaluate restoration of phrenic movement before discharge and during further follow-up visits in case of ongoing PNP. All patients were scheduled for outpatient clinic visits including clinical assessment, echocardiography, 12-lead ECG, and 7-day-Holter-monitoring at 1, 3, and 6 months after the procedure and thereafter every 6 months.

### Statistical Analysis

*t*-test was used to prove differences of numeric values between the two groups if normal distribution with equal variance was given. Normal distribution was determined by Shapiro-Wilk test and equal variance by Brown-Forsythe test. Numeric variables that were not normally distributed were analyzed by Mann-Whitney rank sum test. Categorical variables were analyzed by Chi square test or Fisher's exact-test. A *p*-value < 0.05 was considered significant. Independency of co-variables potentially influencing PNP incidence was proven by uni- as well as multivariate logistic regression. Parameters with a *p*-value of 0.2 or lower in univariate logistic regression were included in multivariate logistic regression. Statistical assessment was performed with Excel (Version 2016, Microsoft Inc., Redmond, WA), XLStat software (V 2016.02.28430, Addinsoft, New York, NY) and SPSS Statistics 25 software (Version 2017, IBM, Armonk, NY, USA). Applied statistical methods were approved by a statistical expert.

## Results

### Study Population

Between 2013 and 2019, we included 632 consecutive AF patients undergoing PVI with the cryoballoon in our study. Mean age was 66.1 ± 10.8 years. 352/632 patients (55.7%) were male and 422 (66.8%) had paroxysmal AF. Mean body mass index (BMI) was 29 ± 6 kg/m^2^. Most common comorbidities were arterial hypertension (503/632 patients, 80%), followed by coronary artery disease (CAD, 224/632 patients, 35%). Mean CHA_2_DS_2_-VAsc score in the study population was 2.7 ± 1.6. Comparison of baseline characteristics between patients with PNP (PNP group) and without PNP (control group, CG) revealed no significant differences and is shown in Table 1.

**Table 1:** Baseline characteristics.

**Baseline characteristics**	**CG**	**PNP**	***p*-value**
*n* (%)	548 (87)	84 (13)	
Age (years), mean ± SD	66 ± 11	66 ± 11	0.75
BMI (kg/m^2^), mean ± SD	29 ± 5	28 ± 7	0.54
Sex (female), *n* (%)	244 (45)	36 (25)	0.77
Paroxysmal AF, *n* (%)	360 (66)	62 (74)	0.15
EHRA-Score, mean ± SD	2.8 ± 0.8	2.8 ± 0.8	0.46
LAD (mm), mean ± SD	46 ± 6	46 ± 7	0.73
LVEF <45%, *n* (%)	76 (14)	10 (12)	0.19
Arterial hypertension, *n* (%)	434 (79)	69 (82)	0.53
Diabetes, *n* (%)	97 (18)	16 (19)	0.76
Coronary artery disease, *n* (%)	195 (36)	29 (35)	0.84
Stroke, *n* (%)	61 (11)	5 (6)	0.25
CHA_2_DS_2_VASc, mean ± SD	2.7 ± 1.6	2.7 ± 1.4	0.87
GFR (ml/min), mean ± SD	65 ± 20	66 ± 20	0.96

*BMI, body-mass-index; EHRA, European Heart Rhythm Association; LAD, left atrial diameter; LVEF, left ventricular ejection fraction; GFR, glomerular filtration rate*.

### Incidence, Distribution, and Type of Phrenicus Nerve Palsy

**Figure 1:**
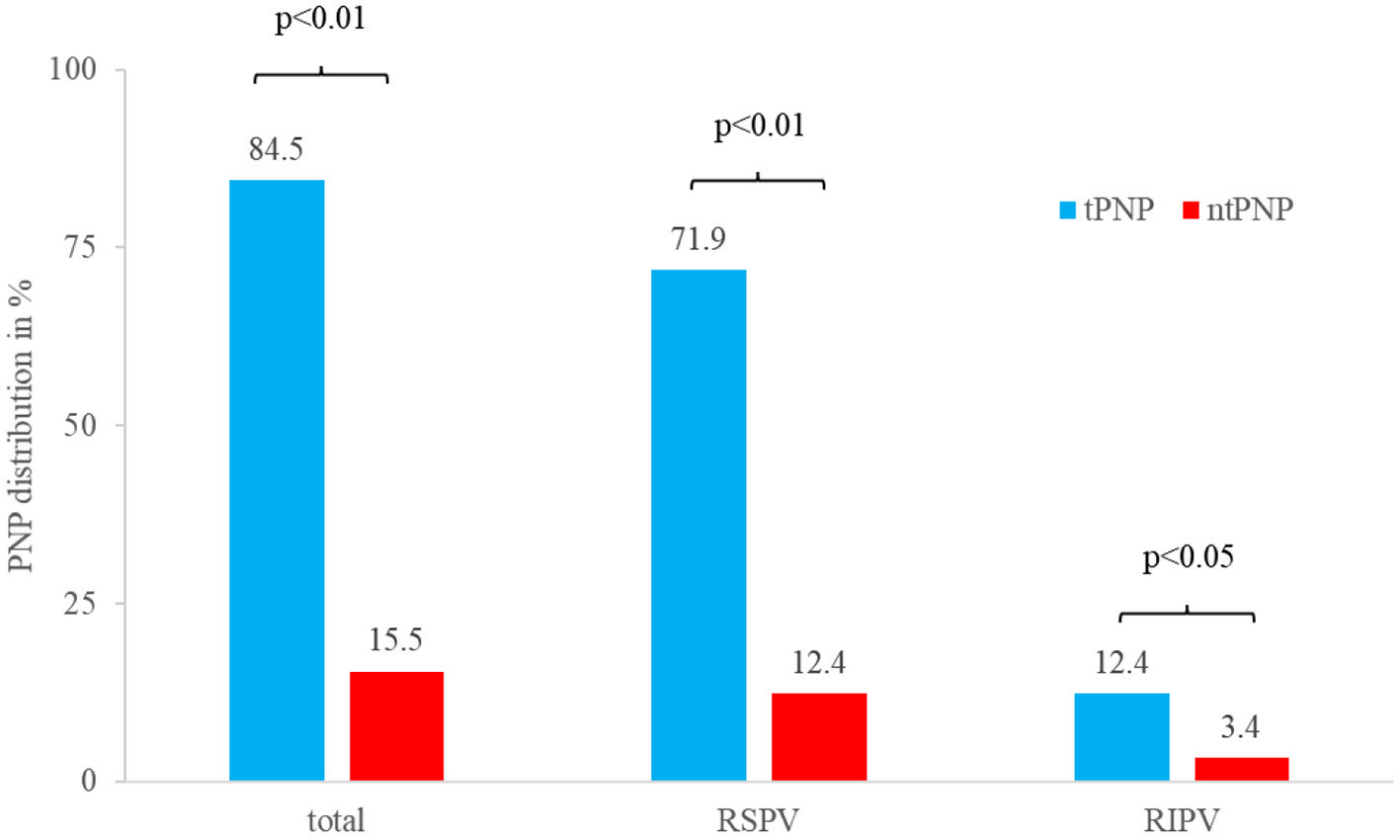
Distribution of tPNP and ntPNP at the right-sided pulmonary veins. Most of the PNP occur during cryoenergy ablation of the RSPV, whereas PNP during ablation of the RIPV is a rare event.

Of 632 consecutive patients undergoing Cryo-PVI 84 patients (13%) experienced PNP during ablation of the right sided PV with five patients having PNP at both the RSPV and the RIPV (total PNP number 89). 75/89 cryoenergy-induced PNP (84%) recovered by the end of the procedure (transient PNP, tPNP), whereas in 14/89 (16%) PNP persisted beyond the end of the procedure (non-transient PNP, ntPNP group). 75/89 PNP (84%) occurred during cryoenergy application at the right superior pulmonary vein (RSPV), whereas 14/89 PNP (16%, *p* < 0.01) were registered during PVI of the right inferior pulmonary vein (RIPV). As seen in Figure 1, the majority of tPNP as well as ntPNP were registered at the RSPV.

### Procedural Data

Mean number of ablations at the RSPV in patients without PNP was 1.6 ± 0.7 and 1.4 ± 0.5 in the ntPNP group, whereas mean number of ablations in the tPNP group was significantly higher (2.2 ± 0.9, *p* < 0.01). Despite higher number of ablations in the tPNP group total freeze duration was comparable to the control group (tPNP: 323 ± 162s vs. CG: 311 ± 171s, *p* = 0.60). In contrast to patients of the control group, total freeze duration in the ntPNP group was significantly shorter (206 ± 135s, *p* = 0.02). Interestingly, minimal balloon temperature (Tmin) in the ntPNP was significantly higher in comparison to Tmin during cryoenergy application without PNP at the RSPV (ntPNP: −45 ± 7°C vs. noPNP: −49 ± 7°C, *p* = 0.04). Accordingly, mean number of ablations at the RIPV in patients with ntPNP was numerically lower and mean freeze duration tend to be shorter compared to RIPV freeze duration without cryoenergy induced PNP. Incidence of both tPNP and ntPNP was not associated with lower isolation rate of the right sided PV (Table 2).

**Table 2:** Procedural data.

**Procedural data**	**noPNP**	**tPNP**	**ntPNP**	***p*-value (No vs. tPNP)**	***p*-value (No vs. ntPNP)**
# RSPV (%)	557 (88)	64 (10)	11 (2)		
# Ablations RSPV	1.6 ± 0.7	2.2 ± 0.9	1.4 ± 0.5	**<0.01**	0.23
Total freeze duration RSPV (s)	311 ± 171	323 ± 162	206 ± 135	0.60	**0.02**
TTI RSPV (s)	40 ± 21	41 ± 26	43 ± 27	0.84	0.83
Tmin RSPV (°C)	−49 ± 7	−52 ± 6	−45 ± 7	**<0.01**	**0.04**
PV isolation rate RSPV (%)	557/557 (100)	63/64 (98)	11/11 (100)	0.97	1.00
# RIPV (%)	618 (98)	11 (2)	3 (<1)		
# Ablations RIPV	1.8 ± 0.9	2.5 ± 1.5	1.3 ± 0.6	0.21	0.27
Total freeze duration RIPV (s)	348 ± 208	365 ± 232	173 ± 57	0.79	0.1
TTI RIPV (s)	48 ± 27	43 ± 6	68 ± 58	0.71	0.65
Tmin RIPV (°C)	−47 ± 9	−48 ± 8	−47 ± 6	0.63	0.96
PV isolation rate RIPV	617/618 (100)	11/11 (100)	3/3 (100)	0.98	1.00

*RSPV, right superior pulmonary vein; RIPV, right inferior pulmonary vein; TTI, time-to-isolation; Tmin, minimal balloon temperature; bold values represent values of statistical significance*.

Remarkably, procedural parameters such as number of ablations until PNP, freeze duration until PNP or balloon temperature at PNP were not different in the tPNP and ntPNP group. However, freeze duration until ntPNP was numerically higher compared to freeze duration until tPNP in both the RSPV and RIPV, suggesting that ntPNP might be associated with longer cryoenergy application (Supplementary Table 1).

### Patient Specific Predictors of Non-transient PNP

To evaluate whether patient-specific constitutional parameters are associated with higher risk of ntPNP during CB-PVI, we compared baseline characteristics that might influence either cryoenergy tissue conduction (e.g., BMI and LAD) or phrenic nerve function such as diabetes or previous stroke in patients with and without ntPNP. Interestingly, we found that incidence of ntPNP is significantly higher in female patients with lower BMI and small atrial size compared to patients without ntPNP (Table 3). Uni-and multivariate regression analysis revealed that sex and BMI are strong and independent risk predictors for ntPNP. Remarkably, female patients have an Odds ratio of 3.9 (95%-CI: 1.1–14.8; *p* = 0.04) to experience ntPNP during CB-PVI compared to male patients (Table 4).

**Table 3:** Constitutional Parameter.

**Constitutional parameters**	**No ntPNP**	**ntPNP**	***p*-value**
BMI (kg/m^2^), mean ± SD	29 ± 6	24 ± 4	**<0.01**
Sex (female), *n* (%)	270 (44)	10 (77)	**0.02**
Paroxysmal AF, *n* (%)	413 (67)	11 (85)	0.24
LAD (mm), mean ± SD	46 ± 7	42 ± 6	**0.04**
Diabetes, *n* (%)	112 (18)	1 (8)	0.48
Stroke, *n* (%)	64 (10)	2 (15)	0.46
CHA_2_DS_2_VASc-Score, mean ± SD	2.7 ± 1.6	3.5 ± 0.9	0.21

*BMI, body-mass-index; LAD, left atrial diameter; bold values represent values of statistical significance*.

**Table 4:** Logistic regression.

**Predictor**	***p*-value:**	***p*-value:**	**Odds**	**CI**
	**univariate**	**multivariate**	**ratio**	**(95%)**
Female sex	0.03	**0.04**	**3.9**	**1.1–14.8**
BMI	<0.01	**0.01**	**0.8**	**0.7–0.9**
LAD	0.02	0.12	0.9	0.85–1.0

*BMI, body-mass-index; LAD, left atrial diameter; bold values represent values of statistical significance*.

### Long-Term Clinical Outcome of Patients With Non-transient PNP

**Figure 2:**
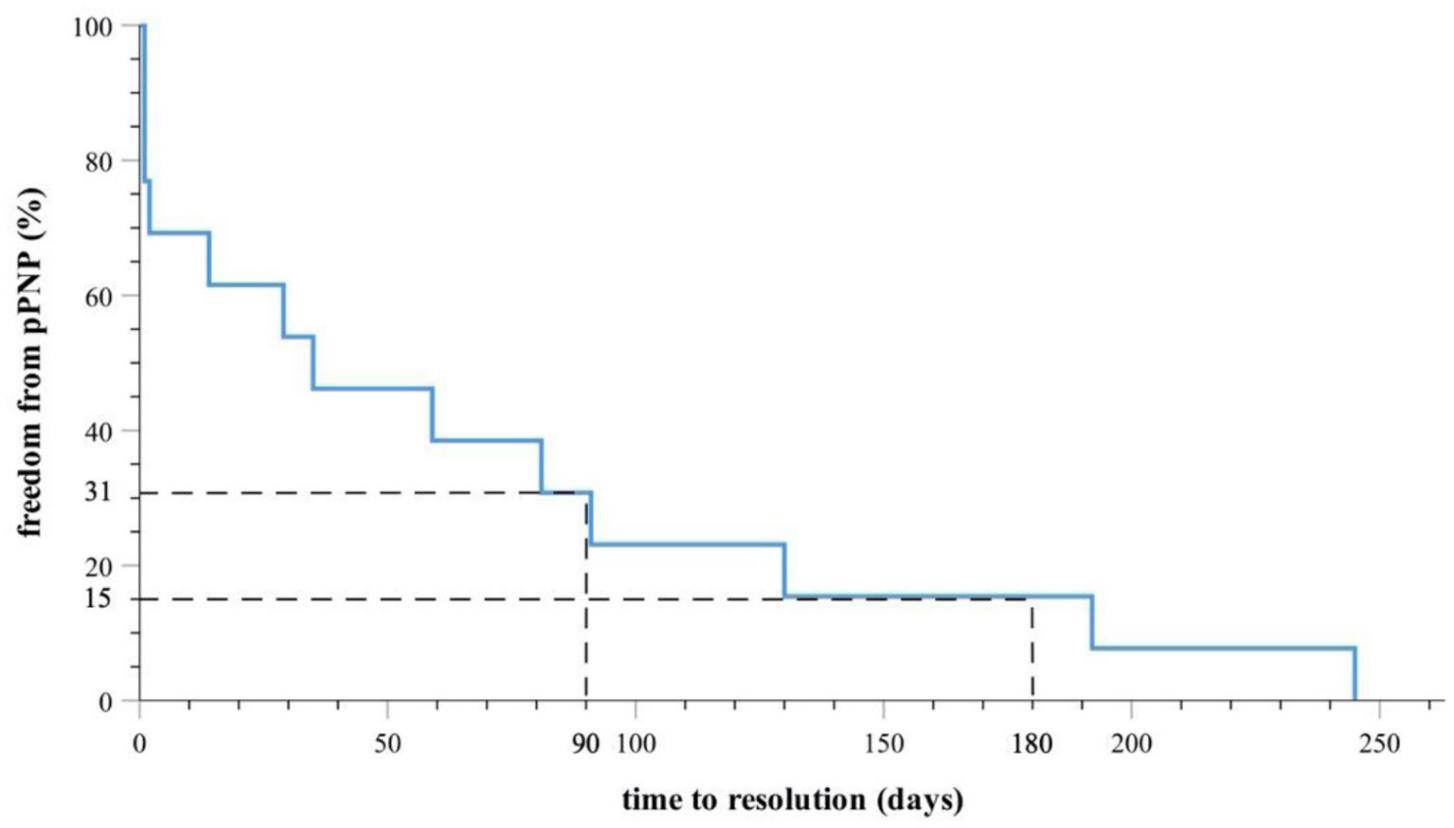
Kaplan-Meier-Survival-Curve indicates complete PNP recovery in all patients during follow-up. Thirty-one percentage of the patients suffered from ntPNP 90 days and 15% of the patients suffered from ntPNP 180 days after index procedure.

All patients with non-transient PNP (14/14, 100%) experienced complete reconstitution of phrenic nerve function after a mean recovery time of 2.2 ± 2.6 months (67.8 ± 79 days, median 35 days). Three months after index procedure in only 2/14 patients (14%) ntPNP was still documented. Longest period of ntPNP reconstitution was 8.2 months (245 days, Figure 2). Symptomatic ntPNP was registered in two patients, which suffered from mild dyspnoea in absence of AF recurrence. Remarkably, symptoms disappeared in these patients after reconstitution of phrenic nerve function.

## Discussion

The present study investigated the correlation between procedural parameters, patient specific conditions and the incidence of phrenicus nerve injury, which is a common and typical procedural complication in AF patients undergoing cryoballoon PVI. Furthermore, long-term outcome and time to recovery of patients, which experienced non-transient PNP, was analyzed.

### Impact of Procedural Characteristics on Incidence and Type of PNP

Remarkably, comparison of procedural data of cryoballoon PVI between patients with tPNP and ntPNP revealed no significant differences, indicating that the incidence of transient or non-transient phrenic nerve injury cannot be exclusively explained by intensity of cryoenergy application.

However, we found several differences between patients with and without PNP. Interestingly, mean number of freeze applications during RSPV isolation was not different in patients without PNP and in patients with ntPNP, suggesting that thermal induced PNP is not a cryoenergy accumulation effect by high number of freezes. This finding is well in line with the fact, that the vast majority of ntPNP during RSPV ablation occurred during the first freeze.

Despite a similar number of cryoenergy applications at the RSPV in patients with ntPNP and without PNP, total freeze duration was significantly shorter and mean nadir balloon temperature was significantly higher in the ntPNP group. These findings reflect the fact, that in patients with ntPNP no further cryoenergy applications are performed at the PV, in which the PNP occurred. Hence, less negative balloon temperature and shorter total freeze duration at the RSPV are a consequence of PNP and do not contribute to PNP genesis.

Since number of freezes, freeze duration or nadir balloon temperature was not different or even less extensive in patients with ntPNP compared to patients with tPNP and without PNP, we conclude that procedural parameters such as number of freezes, freeze duration or nadir balloon temperature are not suitable for PNP prediction during cryoballoon PVI.

### Female Sex and Lower Body-Mass-Index as Independent Predictors of Non-transient PNP

In our study cohort, baseline characteristics between patients with and without PNP did not differ significantly. However, comparing patients with clinically relevant phrenic nerve palsy, defined as PNP holding beyond the end of the procedure, revealed that patients with non-transient PNP are mostly female, have smaller left atrial size and lower BMI compared to patients without ntPNP. Accordingly, uni- as well as multivariate regression identified sex and BMI as strong and independent predictors of ntPNP during cryoenergy ablation of the right-sided veins.

Cryoenergy distribution from the cryoballoon to the phrenic nerve depends on the physical phenomenon that energy transfers from tissue with low temperatures to tissue with higher temperature, so called convective cooling. As a result, cold-induced ice-crystal formation as well as derangement of myocardial microcirculation occurs in atrial cardiomyocytes surrounding the cryoballoon leading to severe and mostly irreversible tissue damage (20).

The cryoenergy flow strongly depends on the distances between tissues with different temperature as well as on the amount of isolating fatty tissue in between. Despite there are no distinct studies evaluating the interorganic distance between rPN and right-sided PV in the context of AF ablation procedures, one might imagine that in females, which have significant lower chest volumina compared to males (21), rPN and left atrium are in close proximity.

### Is There a Protecting Role of Epicardial Fatty Tissue Against Thermal-Induced Phrenic Nerve Injury During Cryoenergy Application?

Epicardial fatty tissue is related to the extent of visceral fatty tissue, rather than to overall adiposity, as shown in several studies (22). Hence, male AF patients, which are more affected from visceral fat compared to female AF patients, might be protected from cryoenergy induced PNP due to the higher extent of isolating epicardial fat tissue. Hence, we conclude that constitutional parameters such as body weight, height and extent of fatty tissue are important factors for incorporal cryoenergy distribution during cryoballoon PVI.

Despite the fact, that our study is non-randomized and data were collected only from one EP center, we propose as a clinical consequence, that female patients with lower BMI should be informed individually about a higher risk of ntPNP during cather ablation using the cryoballoon. Furthermore, in this special patient group, diaphragmatic cMAP monitoring is the method of choice and should be used for the prevention of cryothermal induced PNP in female patients with lower BMI.

## Data Availability Statement

The raw data supporting the conclusions of this article will be made available by the authors, without undue reservation.

## Ethics Statement

The studies involving human participants were reviewed and approved by the Ulm University Medical Center Institutional Review Committee (342/16 and 05/2017). The patients/participants provided their written informed consent to participate in this study.

## Author Contributions

AP: conceptualization, data curation and analysis, methodology, project administration, and writing—review and editing. TD: supervision, conceptualization, project administration, and writing—review and editing. WR: supervision. SM, DB, MB, and KW: data analysis and writing—original draft preparation. CB: data analysis. CS, YT, and HW: data curation and analysis and methodology. All authors contributed to the article and approved the submitted version.

## Conflict of Interest

TD received speaker's honoraria and consulting fees from Medtronic, Biosense Webster, Boerhringer-Ingelheim, Bayer, Daiichi-Sankyo. AP received speaker's honoraria from Medtronic, Biosense Webster, Daiichi-Sankyo and is invited fellow of the Boston Scientific EP training program. CS and YT were funded by the Deutsche Herzstiftung. The remaining authors declare that the research was conducted in the absence of any commercial or financial relationships that could be construed as a potential conflict of interest.

## Publisher's Note

All claims expressed in this article are solely those of the authors and do not necessarily represent those of their affiliated organizations, or those of the publisher, the editors and the reviewers. Any product that may be evaluated in this article, or claim that may be made by its manufacturer, is not guaranteed or endorsed by the publisher.
