# Supplementary material for: Predicting Phrenic Nerve Palsy in Patients Undergoing Atrial Fibrillation Ablation With the Cryoballoon—Does Sex Matter?
Source: Front Cardiovasc Med. 2021 Dec 14;8:746820. doi: 10.3389/fcvm.2021.746820 (PMC8712427; doi:10.3389/fcvm.2021.746820)
Supplement: Supplementary file 1 [file Table_1.docx]

Supplementary table 1:

| **Procedural data** | **tPNP** | **ntPNP** | **p-Value** |
| --- | --- | --- | --- |
|  |  |  |  |
| **RSPV** |  |  |  |
| n (%) | 64 (85) | 11 (79) | 0.62 |
| # ablations until PNP at RSPV | 1.3 ±0.4 | 1.1 ±0.3 | 0.25 |
| Freeze duration until PNP [sec] | 113 ±55 | 135 ±46 | 0.20 |
| T_PNP_ [°C] RSPV | -47 ±11 | -44 ±6 | 0.23 |
| PV-Isolation rate at PNP | 48 (75) | 11 (100) | 0.11 |
|  |  |  |  |
| **RIPV** |  |  |  |
| n (%) | 11 (79) | 3 (21) | 0.45 |
| # ablations until PNP at RIPV | 1.2 ±0.6 | 1.0 ±0.1 | 0.60 |
| Freeze duration until PNP [sec] | 131 ±59 | 201 ±60 | 0.16 |
| T_PNP_ [°C] RIPV | -47 ±6 | -49 ±4 | 0.55 |
| PV-Isolation rate at PNP | 8 (73) | 3 (100) | 1.00 |
| RSPV: right superior pulmonary vein; RIPV: right inferior pulmonary vein; PNP: phrenic nerve palsy | | | |
